# The Role of Resilience in the Relationship Between Cyberbullying and Depression, Anxiety, and Stress in Adolescents

**DOI:** 10.1002/brb3.70916

**Published:** 2025-10-20

**Authors:** Zafer Korkmaz, İlhan Çiçek, Metin Buluş, Mehmet Emin Şanlı, Murat Yıldırım

**Affiliations:** ^1^ Faculty of Education Van Yüzüncü Yıl University Van Turkey; ^2^ Health College Batman University Batman Turkey; ^3^ Faculty of Education Adıyaman University Adıyaman Turkey; ^4^ Health Vocational School Batman University Batman Turkey; ^5^ Department of Psychology, Faculty of Science and Letters Ağrı İbrahim Çeçen University Ağrı Turkey; ^6^ Psychology Research Center Khazar University Baku Azerbaijan

**Keywords:** adolescents, anxiety, cyberbullying, depression, resilience, stress

## Abstract

**Purpose:**

This study investigates the mediating role of resilience in the relationship between cyberbullying and depression, anxiety, and stress.

**Method:**

Data were collected from 591 adolescents (51.6% boys; *M*_age_ = 15.77 ± 1.12) using a socio‐demographic questionnaire, the Cyberbullying Scale, the Brief Resilience Scale, and the Depression, Anxiety, and Stress Scale (DASS‐21).

**Finding:**

Bivariate correlations revealed a moderate positive association between cyberbullying, anxiety, and stress. Structural equation modeling indicated that higher levels of cyberbullying were significantly associated with increased anxiety and stress but not with depression. Resilience was found to partially mediate the relationship between cyberbullying and stress but did not mediate the relationships between cyberbullying and either depression or anxiety.

**Conclusion:**

These findings highlight that cyberbullying is a strong predictor of anxiety and stress, with resilience playing a key role in mitigating stress levels. Interventions focused on building resilience may offer an effective strategy for reducing stress among adolescents experiencing cyberbullying.

## Graphical abstract

This study explored how cyberbullying affects teenagers’ mental health, focusing on depression, anxiety, and stress, and whether resilience helps protect them from these negative effects. Researchers surveyed 591 adolescents and found that those who experienced more cyberbullying tended to feel more anxious and stressed, though not necessarily more depressed. Interestingly, resilience—the ability to recover from challenges—played a mitigating role by reducing the impact of cyberbullying on stress. However, it didn't seem to help with anxiety or depression. These findings suggest that while cyberbullying can seriously affect young people's emotional well‐being, especially by increasing stress and anxiety, building resilience may be a useful strategy to help them cope better with stressful experiences online. Supporting adolescents in developing resilience could be an important part of school‐based or community interventions aimed at reducing the harmful effects of cyberbullying.

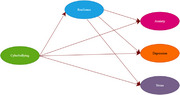

## 1 Introduction

The concept of cyber has evolved into a phenomenon that emerged with the development of the internet and the advancement of technology, influenced by the internet's evolution, where users interact in the digital world (Tomasello 2023). Today, cyberspace is a vast and indispensable system that enables users to perform various activities online. Cyberspace applications, which are diversifying and becoming widespread with the development of technology, create some areas of conflict among people. This conflict in the cyber world emerges as two important concepts, such as “bullying” and “cyberbullying” (CB) (Keller 2012). In general, the term “bullying” can be defined as the act of a group or individual harming or attacking another group/individual physically, psychologically, socially, or verbally (Garaigordabil and Martinez‐Valderrey 2018).

With the dissemination of technological communication tools in cyberspace, conflicts in cyberspace can sometimes reach the level of bullying. CB refers to intentional, aggressive, and repetitive behaviors carried out in digital environments, such as the Internet, social media, and digital communication tools. There are different forms of CB, such as sending threatening messages, making derogatory or humiliating comments, exhibiting manipulative behavior, spreading false information, and disclosing private information (Olweus and Limber 2018). Besides, CB is similar to physical bullying and has become more common and damaging with its features such as anonymity and the ability to reach large audiences (Hinduja and Patchin 2017); thus, CB is becoming an increasingly serious problem, especially among adolescents.

Adolescence is also a phase in which physical and emotional changes occur rapidly, and this process is considered a critical and sensitive period for adolescents (Çiçek 2020). In recent years, with the deployment of the internet and social media platforms, adolescents have encountered CB more frequently in digital environments (Santre 2023). In a study by Wang et al. (2025), it was reported that 28.3% of students had been subjected to bullying, with 23.2% experiencing traditional bullying, 17.7% experiencing CB, and 8.1% experiencing cyberattacks. Students who experienced all three types of bullying had a significantly higher risk of insomnia (4.89 times higher), anxiety (ANX; 11.42 times higher), depression (DEP; 11.52 times higher), and PTSD (15.48 times higher). CB continues to be a prevalent issue, especially among young people. According to a study conducted by the World Health Organization (WHO), one in six adolescents is exposed to CB, with rates of 16% in girls and 15% in boys (Euronews 2024). The (İmdat 2021) found that 28% of students aged 14–19 had engaged in CB, while 30% had been victims of it. The same report referenced a 2017 study by Samsung Turkey and the BTK, which indicated that 11.64% of students had experienced CB. Additionally, UNICEF's 2019 report showed that 70.6% of young people aged 15–24 had been subjected to online violence, CB, or digital harassment (İmdat 2021). According to XNSPY Blog's 2025 statistics, approximately 46% of US adolescents aged 13–17 have been victims of CB at some point in their lives. In 2023, 26.5% of this age group reported experiencing CB in the past 30 days. The lifetime exposure rate was recorded at 59.2% for girls and 49.5% for boys (XNSPY 2025).

Studies highlight that adolescents exposed to CB have an increased risk of encountering serious problems such as emotional problems, low self‐esteem, ANX, DEP, and suicidal thoughts (Hinduja and Patchin 2017; Xu et al. 2023). Additionally, adolescents exposed to CB often appear to be the target of aggressive behavior such as threats, insults, humiliation, or manipulation, and such attacks can lead to psychological problems such as DEP, ANX, and stress (STR) in adolescents (Olweus and Limber 2018; Xu et al. 2023).

One of the most common effects of CB in adolescents is DEP, which is defined as an emotional disorder and mental health problem, and it may lead to individual, social, professional, and economic losses and serious health problems in human beings’ lives (Compas et al. 1993). This psychological problem manifests itself in individuals with situations such as feelings of worthlessness, helplessness, hopelessness, and pessimism; feelings of guilt; ANX; concentration and speech difficulties; obsessive thoughts; suicide attempts; thoughts of death; extreme weakness, social media use, eating disorders, and sleep disorders (Çi̇çek et al. 2023; Korkmaz et al. 2023). Adolescents engaged in CB may experience symptoms of DEP such as hopelessness, helplessness, low self‐esteem, loss of interest, and suicidal thoughts. Similarly, investigations reveal that CB increases the risk of DEP, which can lead to long‐term negative effects (Hinduja and Patchin 2017; Santos et al. 2021).

ANX, which is associated with DEP and occurs when an undesirable situation is encountered, is likely to be observed in cyberbullied people (Chu et al. 2018). ANX is a complex structure including physiological, emotional, and behavioral responses and is generally a lack of concentration, sleep disorders, an inability to avoid bad thoughts, and an uneasy mood. It also manifests itself with symptoms such as weakness in coping with STR and problem‐solving skills, emotional instability, a pessimistic perspective, deterioration in general relations with the environment, avoidance of close relationships, and perceiving the environment as a threat (APA 2013; Gu et al. 2010; Viana et al. 2018). Adolescents subjected to CB may experience ANX symptoms such as social phobia, panic attacks, ANX, and post‐traumatic stress disorder (PTSD). Constantly feeling threatened in digital environments, fear of social rejection, and exposure to criticism may increase ANX levels in adolescents (Q. Li et al. 2019).

The concept of STR, which is rather crucial in people's agendas both individually and socially, may be defined as the ongoing sensation of spiritual strain coming with human relationships becoming more intense and complex in today's modern society. (Lazarus 1966). In other words, there is a triggering situation caused by pressure, expectation, or threat. Thus, the individual's ability to fight for the perception of her/his adequacy is negatively affected. An increase in STR levels may also be considered one of the consequences of CB on adolescents (Garaigordobil and Machimbarrena 2017). Adolescents exposed to CB may face a persistent STR response, and this case may manifest itself as physical symptoms (headache, stomach upset), emotional symptoms (irritability, restlessness), and cognitive symptoms (difficulty concentrating, forgetfulness) (González‐Cabrera et al. 2017; Alhujailli et al. 2020). CB is a source of STR affecting the daily lives of adolescents. Victim adolescents may feel under constant threat in digital environments, which can lead to consequences such as shyness, isolation, and feelings of insecurity in social relationships.

Individuals' reactions to events or coping strategies for these events differ from each other; some people may experience situations challenging daily life, such as DEP, ANX, and STR, and the negative mood occurring in stressful and traumatic cases may last for a very long time. (Tusaie and Dyer 2004). On the other hand, some people can get out of the mood caused by such negative experiences in a very short time and return to their normal lives very quickly. The ability of individuals to recover from negative events and quickly return to their normal lives is explained by the concept of resilience (PR) in the positive psychology approach (Aliyev and Gengec 2019; B. W. Smith et al. 2008). PR is the power to resist, cope, recover, and succeed in the face of destructive and negative life experiences (Den Hartigh and Hill 2022). In other words, PR is the individual's ability to successfully overcome negative conditions, adapt to new situations, and resist and be successful in the face of negative life experiences (Bulut et al. 2013; Den Hartigh and Hill 2022; Gizir 2007). A negative relationship has been found between PR and destructive variables, such as STR, ANX, DEP, and smartphone addiction (Anyan and Hjemdal 2016; Santos et al. 2021; Şanli et al. 2023; Yıldırım and Çiçek 2022; Yıldırım and Green 2024). It was determined that adolescents with high levels of PR had lower DEP and ANX scores (Hjemdal et al. 2011). Other studies have also found that PR plays an important role in both preventing and coping with disorders, such as DEP, ANX, and STR (Bayat and Olca 2023; Ozawa et al. 2017). Anderson et al. (2022) conducted a study on adolescents and asserted that PR has a mediating role between bullying, DEP, and ANX, and PR can be seen as a protective factor against bullying.

In conclusion, it is believed that PR may play a critical mediating role in alleviating the negative mental health effects of CB. This mechanism reduces the traumatic impact by allowing individuals to personalize, make sense of, and develop effective coping strategies such as active problem‐solving or seeking social support in response to stressful events (such as cyberattacks) (Machmutow et al. 2012; Zhou et al. 2013). It has been observed that individuals with high levels of PR exhibit significantly lower levels of psychological distress and perceive these experiences as less stressful, even when they are victims of CB (Lin et al. 2022; Hinduja and Patchin 2019). This suggests that PR filters the direct negative effects of CB by enhancing an individual's ability to cope with STR and helps maintain psychological well‐being.

### 1.1 Present Study

CB has become a significant and growing concern, particularly among adolescents, where its prevalence and severity continue to rise (Kurt et al. 2023; Santre 2023; Xu et al. 2023). Understanding the psychological impact of CB on adolescents’ mental health and identifying mitigating factors that can mitigate its adverse effects are critical for promoting mental health and well‐being. Therefore, this study aims to investigate the role of PR as a buffer against the negative psychological impacts of CB, focusing on its potential to reduce symptoms of DEP, ANX, and STR among adolescents. By examining the associations between CB and adolescent mental health, this research aims to contribute to the development of targeted interventions and preventive strategies that build PR, ultimately helping to reduce the mental health challenges caused by CB. The primary objective of this study is to examine the mediating role of PR in the relationship between CB and DEP, ANX, and STR. To achieve this, the study tests the following hypotheses:

**H1**: *There is a negative relationship between cyberbullying and resilience*.

**H2**: *There is a positive relationship between cyberbullying and depression, anxiety, and stress*.

**H3**: *Resilience mediates the relationship between cyberbullying and depression, anxiety, and stress*.

## 2 Method

### 2.1 Participants and Procedure

A total of 591 adolescents, 286 (48.4%) girls and 305 (51.6%) boys, participated in the study. The ages of the participants ranged from 13 to 18 (*M* = 15.77). The study group was determined by the appropriate sampling method, and the study data were collected face‐to‐face from high school students in a region located in southeastern Turkey between March 15 and April 10, 2023. The criterion for inclusion in the study was that the adolescent should be enrolled in high school. Before proceeding with data collection, ethics committee permission was obtained from Batman University (2023/02‐34). Consent was also obtained from the students' parents before implementation, as students were informed about the study, and they were informed that participation was voluntary and the data obtained would be used for only scientific purposes.

### 2.2 Measures

#### 2.2.1 Depression Anxiety Stress Scale

The Depression Anxiety Stress Scale (DASS‐21), developed by Lovibond and Lovibond (1995), consists of 21 items and has a 4‐point Likert‐type rating. The reliability and validity study of the short form of the scale was conducted by Henry and Crawford (2005). The Turkish adaptation was made by Yılmaz et al. (2017). Scores from the scale vary between 7 and 28. High scores on the scale indicate high levels of DEP, ANX, and STR.

#### 2.2.2 Brief Resilience Scale

The scale was developed by B. W. Smith et al. (2008). The scale measures the PR levels of individuals. The Brief Resilience Scale (BRS) is a 5‐point Likert‐type, 6‐item, self‐report measurement tool. High scores on the scale indicate high psychological PR. The Turkish adaptation of the scale was made by Doğan (2015). In the adaptation study, the internal consistency coefficient was found to be 0.83.

#### 2.2.3 Cyberbullying Scale

The scale was adapted by Stewart et al. (2014). The scale, which is a 5‐point Likert‐type scale, consists of 14 items. The scores on the scale vary between 0 and 60. The Turkish adaptation of the scale was made by Küçük (2016). High scores on the scale indicate that individuals are exposed to high levels of CB.

### 2.3 Analytic Approach

We estimated the sample size using the pwrss R package (Bulus 2023a, 2023b; Bulus and Polat 2023), aiming to detect an indirect effect as small as 0.04, considering Sobel's *z* test with a type I error rate of 5% and power rate of 80%. The minimum required sample size was determined to be 393. Eventually, the data was collected from 591 participants within the budget. Data analysis was conducted in multiple stages using various R packages in the R environment (R Core Team 2022), such as psych (Revelle 2019), semTools (Jorgensen et al. 2018), and lavaan (Roessel 2012). Descriptive statistics were examined through the psych package, while scale reliabilities were assessed via semTools. Confirmatory factor analysis (CFA) models and the hypothesized structural mediation model were tested using the lavaan package, with diagonally weighted least squares (DWLS) estimation, which considers ordinal indicator variables.

## 3 Results

### 3.1 Preliminary Analysis

Table 1 presents descriptive statistics. The data were collected from adolescents in high school, ranging in age from 13 to 18 years old (mean age = 15.77). Among the 591 observations, 48.4% were girls, whereas 51.6% were boys. A total of 80% of them have a social media account, and they engage in social media slightly more than 2 h a day. Overall, they had low levels of STR and moderate levels of ANX and DEP.

**TABLE 1:** Descriptive Statistics.

Variable	Mean	SD	Median	Min	Max	Skew	Kurtosis
Age	15.77	1.12	16	13	19	−0.21	−0.34
Gender	1.52	0.50	2	1	2	−0.07	−2.00
Has smartphone	1.42	0.49	1	1	2	0.31	−1.90
Has a social media account	1.20	0.40	1	1	2	1.52	0.30
Time spent on social media	2.19	0.98	2	1	4	0.34	−0.93
Resilience	17.36	4.17	18	6	30	−0.36	0.79
Cyberbullying	13.14	10.46	11	0	52	0.81	0.02
Anxiety	13.19	4.23	13	7	27	0.45	−0.43
Depression	14.63	4.62	14	7	28	0.21	−0.73
Stress	14.26	4.53	14	7	28	0.17	−0.78

*Note: N* = 552. Has a smartphone (1: yes, 2: no). Has a social media account (1: yes, 2: no). Gender (1: female, 2: male). Time spent on social media (1: 1 h in a day, 2: 1–2 h in a day, 3: 3–5 h in a day, 4: more than 6 h in a day).

**FIGURE 1:**
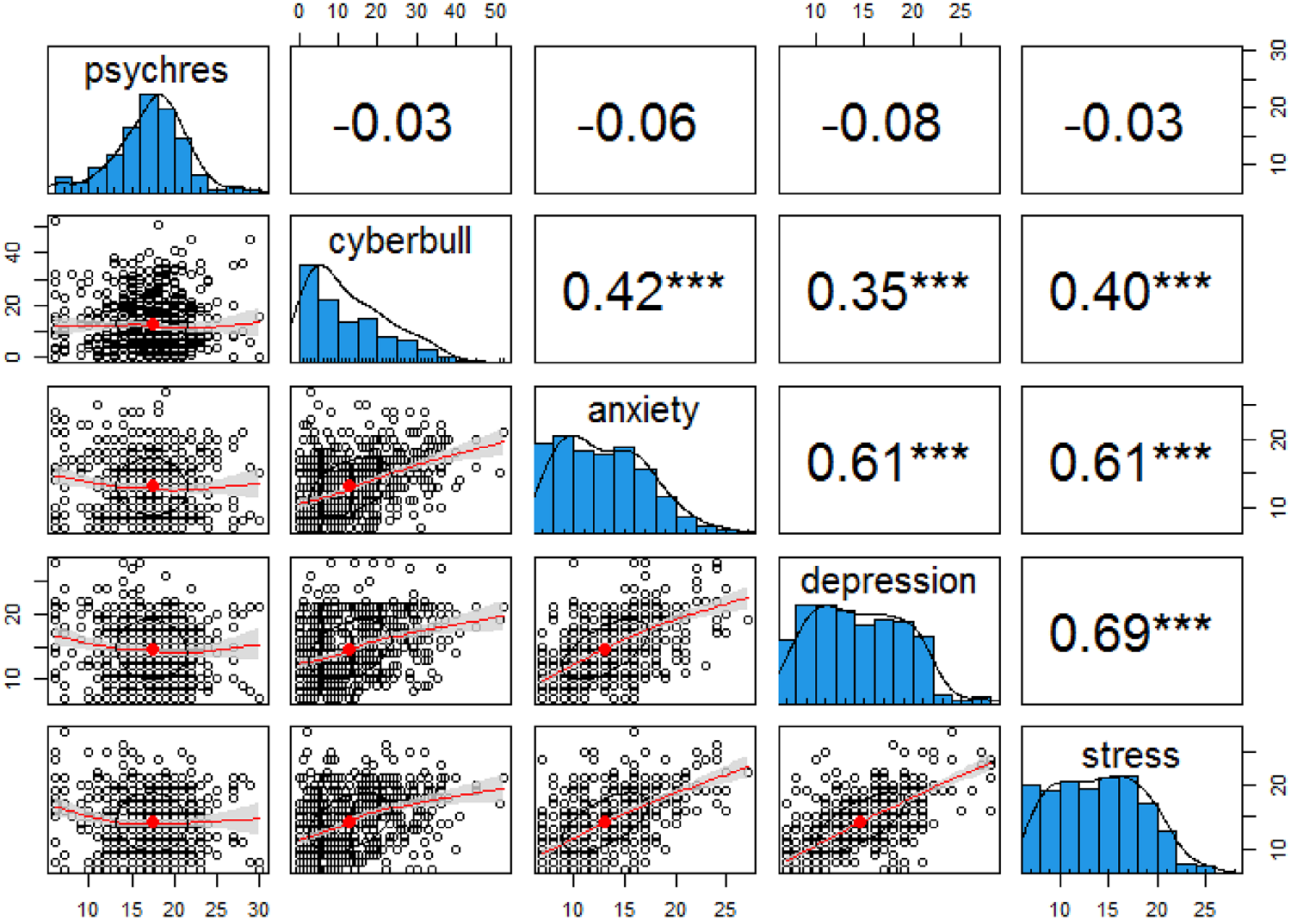
Spearman's rho correlation coefficients between variables of interest.

Bivariate correlations between variables of interest are presented in Figure 1. We interpreted correlation coefficients in line with benchmarks presented by Cohen (1988). There were positive large correlations among the ANX–DEP–STR triad. This is expected, as they are falling under the same scale. CB had a positive moderate correlation with the ANX–DEP–STR triad. This means CB is a potentially strong predictor of the triad.

The correlation between PR and CB was negative and very small. The correlation between PR and the triad was also negative and small. Some evidence made us believe that the true correlation between PR and the ANX–DEP–STR triad may be attenuated for several reasons. This issue will be discussed further in the following paragraphs.

### 3.2 Measurement Model

Table 2 presents unidimensional CFA model fit indices, Cronbach's α reliability coefficient suitable for ordinal scales (Zumbo et al. 2007), and average variance extracted for each of the PR, CB, ANX, DEP, and STR scales. CFA models were evaluated using the chi‐square statistic ($\chi^{2}$), degrees of freedom (df), $\chi^{2}/\mathrm{df}$ ratio, comparative fit index (CFI), Tucker–Lewis Index (TLI), root mean square error of approximation (RMSEA), and standardized root mean square residual (SRMR).

**TABLE 2:** Measurement models.

	Fit							Reliability	
Variable	$\chi^{2}$	df	$\chi^{2}$/df	CFI	TLI	RMSEA	SRMR	α	AVE
Resilience	7.54	6	1.26	1.00	1.00	0.02	0.02	0.54	0.16
Cyberbullying	178.89	77	2.32	0.99	0.99	0.05	0.05	0.92	0.48
Anxiety	24.52	14	1.75	1.00	0.99	0.04	0.04	0.84	0.45
Depression	18.88	14	1.35	1.00	1.00	0.03	0.03	0.88	0.52
Stress	43.50	14	3.11	0.99	0.99	0.06	0.04	0.85	0.46

Abbreviations: AVE, average variance extracted; CFI, comparative fit index; RMSEA, root mean square error of approximation; SRMR, standardized root mean square residual; TLI, Tucker–Lewis index.

The PR CFA model had a $\chi^{2}/\mathrm{df}$ ratio of 1.26, the CB CFA model had a $\chi^{2}/\mathrm{df}$ ratio of 2.32, the ANX CFA model had a $\chi^{2}/\mathrm{df}$ ratio of 1.75, the DEP CFA model had a $\chi^{2}/\mathrm{df}$ ratio of 1.35, and the STR CFA model had a $\chi^{2}/\mathrm{df}$ ratio of 3.1:1 indicating an acceptable fit. All models had CFI ≥ 0.99, TLI ≥ 0.99, RMSEA ≤ 0.06 and SRMR ≤ 0.04, indicating excellent model‐data fit. It should be noted that items with negative valence were correlated in the PR CFA model. Without this modification, fit indices were not so good.

The CB, ANX, DEP, and STR scales all had a high Cronbach's alpha α reliability estimate (≥ 0.84). The CFA models for CB, ANX, DEP, and STR scales extract ≥ 45% of the variation in their observed items. However, surprisingly, the PR scale had a Cronbach's alpha of 0.54, which is relatively low compared to other scales. The extracted variance was merely 16%. There are some potential culprits. The scale was adapted initially using a sample of college students (ATIF). Some studies report relatively low‐reliability estimates for high school students. Thus, it could be that the PR scale may not be suitable for adolescents. Another explanation for low reliability could be that the scale consists of three items with negative valence and three items with positive valence. Some research indicates that mixed item format produces lower reliability estimates for unidimensional constructs (Salazar 2015). We decided to move forward because some research suggests a reliability estimate of around 0.50 can be satisfactory for theory building but not for high‐stakes testing (Taber 2018).

In conclusion, the measurement models for PR, CB, ANX, and DEP had a good to acceptable fit to the data, indicating that these models are reliable and valid measures of their respective constructs. The reliability of the scales for each construct was generally good, except for the PR scale, which had a low alpha. The AVE for each scale was also generally good, with CB having the highest AVE and PR having the lowest. Researchers should consider these findings when selecting measures for these constructs in future studies.

### 3.3 Structural Model

**FIGURE 2:**
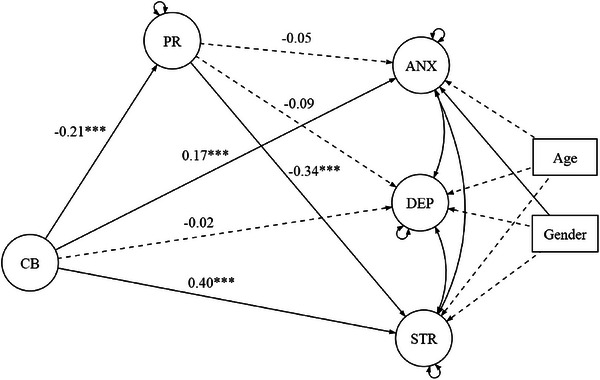
Structural mediation model with standardized path coefficients. Path coefficients that are not statistically significant were marked with intermittent directional arrows.

This report describes the results of a mediation model examining the relationship between CB, PR, and three outcomes: DEP, ANX, and STR. The model also includes age and gender as covariates to adjust for their potential influence on the relationships. The structural mediation model can be seen in Figure 2. Results are presented in Table 3.

**TABLE 3:** Standardized estimates for the structural mediation model.

Effect	Label	Std. β	SE	*z*	*p* Value
CB → PR	a	−0.21	0.05	−4.00	0.000
CB → DEP	$c_{1}^{\prime }$	−0.02	0.04	−0.47	0.637
PR → DEP	$b_{1}$	−0.09	0.05	−1.82	0.069
CB → ANX	$c_{2}^{\prime }$	0.17	0.04	3.98	0.000
PR → ANX	$b_{2}$	−0.05	0.05	−0.92	0.358
CB → STR	$c_{3}^{\prime }$	0.40	0.05	8.65	0.000
PR → STR	$b_{3}$	−0.34	0.06	−5.98	0.000
Indirect CB → PR → DEP	$ab_{1}$	0.02	0.01	1.64	0.101
Total CB on DEP	$ab_{1}+c_{1}^{\prime }$	0.00	0.04	0.02	0.982
Indirect CB → PR → ANX	$ab_{2}$	0.01	0.01	0.89	0.371
Total CB on ANX	$ab_{2}+c_{2}^{\prime }$	0.18	0.05	4.01	0.000
Indirect CB → PR → STR	$ab_{3}$	0.07	0.02	3.21	0.001
Total CB on STR	$ab_{3}+c_{3}^{\prime }$	0.47	0.04	11.37	0.000

Abbreviations: ANX, anxiety; CB, cyberbullying; DEP, depression; PR, resilience; SE, standard error for the standardized path coefficient; Std. β, standardized path coefficient; STR, stress.

The coefficient for the CB → PR path was statistically significant (a = −0.21, SE = 0.05, *p* < 0.001), indicating that CB was negatively associated with PR. However, the coefficient for the PR → DEP path was not statistically significant (*b* = −0.09, SE = 0.05, *p* = 0.069), suggesting that PR did not mediate the relationship between CB and DEP ($ab_{1}$ = 0.02, SE = 0.01, *p* = 0.101). The direct effect of CB on DEP was also not statistically significant ($c_{1}^{\prime }$ = −0.02, SE = 0.04, *p* = 0.637), indicating that CB did not directly affect DEP after accounting for the effect of PR, gender, and age. Finally, the total effect of CB on DEP was not statistically significant ($ab_{1}+c_{1}^{\prime }$ = 0.00, SE = 0.04, *p* = 0.982), indicating that higher scores on CB were not associated with higher DEP after accounting for gender and age.

The coefficient for the PR → ANX path was not statistically significant (*b* = −0.05, SE = 0.05, *p* = 0.358), suggesting that PR did not mediate the relationship between CB and ANX ($ab_{2}$ = 0.01, SE = 0.01, *p* = 0.371). The direct effect of CB on ANX was statistically significant ($c_{2}^{\prime }$ = 0.17, SE = 0.04, *p* < 0.001), indicating that CB was associated with higher scores on ANX after accounting for the effect of PR, gender, and age. Finally, the total effect of CB on DEP was also statistically significant ($ab_{2}+c_{2}^{\prime }$ = 0.18, SE = 0.05, *p* < 0.001), indicating that higher scores on CB were associated with higher ANX scores after accounting for gender and age.

The coefficient for the PR → STR path was statistically significant (*b* = −0.34, SE = 0.06, *p* < 0.001), suggesting that PR mediated the relationship between CB and STR ($ab_{3}$ = 0.07, SE = 0.02, *p* = 0.001). The direct effect of CB on STR was also statistically significant ($c_{3}^{\prime }$ = 0.40, SE = 0.05, *p* < 0.001), indicating that CB was associated with higher scores on STR after accounting for the effect of PR, gender, and age. Finally, the total effect of CB on STR was also statistically significant ($ab_{3}+c_{3}^{\prime }$ = 0.47, SE = 0.04, *p* < 0.001), indicating that higher scores on CB were associated with higher scores on STR after accounting for gender and age.

In conclusion, this mediation model found that CB was associated with ANX and STR but not DEP. PR partially mediated the relationship between CB and STR, but not between CB and DEP or ANX. These findings suggest that interventions aimed at increasing PR may be effective in reducing the negative effects of CB on STR.

## 4 Discussion

The primary aim of the present study is to investigate the mediating role of PR in the relationship between CB and DEP, ANX, and STR. The results of our study revealed no significant relationship between CB and PR, thus failing to support (H_1_). However, a review of the literature indicates the presence of findings that generally contradict our results (Collen and Onan 2021; Güçlü‐Aydogan et al. 2023; Hinduja and Patchin 2017; Navarro et al. 2018). For example, Santos et al. (2021) reported a negative relationship between CB and PR among adolescents, suggesting that PR functions as a protective factor against the adverse mental health effects of CB. Similarly, Batmaz et al. (2022) found a negative association between CB and PR in their study.

The results confirm the second hypothesis (H_2_) of our research, “There is a positive relationship between cyberbullying, depression, anxiety, and stress.” Similar results were found in previous studies (Chu et al. 2018; Cole et al. 2016; Niu et al. 2020; Wright 2015), and Hamm et al. (2015) found a positive relationship between CB and DEP in adolescents. Likewise, Fahy et al. (2016) emphasized that there is a positive relationship between CB and DEP. According to Grigore and Maftei's (2020) research, it was stated that there was a positive relationship between the ANX levels of adolescents exposed to CB. Studies conducted by P. K. Smith et al. (2018) and D. Li et al. (2020) also pinpointed that CB increases ANX levels.

The results obtained from this study partially confirm the third hypothesis of our research (H_3_): “Resilience mediates the relationship between cyberbullying and depression, anxiety, and stress.” The analysis showed that PR only had a mediating role in the relationship between CB and STR. On the other hand, it was determined that PR did not mediate the relationship between CB and DEP and ANX. When the literature is examined, it may be claimed that there are studies partially similar to the results of our study (Şahin and Ayaz 2018). Anderson et al. (2022) found that the relationship between bullying, ANX, and DEP was mediated by PR. Similarly, Lin et al. (2022) revealed that PR has a mediating role in the relationship between CB and DEP. Additionally, the AlShehry et al. (2023) study with university students indicated that PR had a mediating role between CB and DEP. These results reveal the protective aspect of PR. Navarro et al. (2018), in their study on adolescents, found that PR mediates between CB and fatalism. High‐STR levels have been determined in individuals exposed to CB. The result of our study indicates that CB is an important source of STR affecting the daily lives of adolescents. Similar results were obtained in previous studies (Kowalski et al. 2014; Garaigordobil and Machimbarrena 2017; González‐Cabrera et al. 2017; Alhujailli et al. 2020). To better understand the protective role of PR on mental health, it can be explained more comprehensively within the framework of coping models. When faced with STR factors such as CB, resilient individuals tend to perceive threats as less destructive and respond more adaptively. This occurs through mechanisms such as cognitive restructuring, emotional regulation, and problem‐focused coping strategies (D'Zurilla and Nezu 2010). For example, Machmutow et al. (2012) indicated that adolescents who can manage their emotional responses and seek social support are more resilient to depressive symptoms caused by CB. Therefore, PR acts as a mediator that directly reduces the impact of the STR caused by CB on mental health and strengthens the individual's adaptive responses.

In general, the results of the study are consistent with the existing literature. CB has serious and complex negative effects on individuals' mental health, leading to symptoms such as ANX disorders, DEP, loss of self‐esteem, and even PTSD (Kan 2024; Sır Psikoloji 2024; Zhu et al. 2021). Specifically, in adolescents, victimization from CB can increase levels of DEP and negatively affect overall psychological well‐being (Boyar and Arslantaş 2024). However, PR stands out as a significant protective factor against the negative mental health effects of CB (Ulaş 2024; Santos et al. 2021; Onan 2021). PR helps individuals enhance their coping mechanisms for bullying experiences, improving their psychological adjustment and reducing negative outcomes. In this context, the findings of the study emphasize the importance of not only preventive measures against bullying but also interventions that support individuals' mental health and enhance their PR in combating CB.

### 4.1 Limitations

In this study, the relationships between CB and PR, DEP, ANX, and STR were examined, and although important findings were reached, there are some limitations. First, the age and geographic distribution of participants in the study sample are limited, which may limit generalizability. The measurement method, in which participants self‐report, may affect the reliability of subjective evaluations, and we may have difficulties establishing cause‐and‐effect relationships. Additionally, since the study had only a cross‐sectional design, it cannot evaluate changes over time and long‐term effects. This limits the opportunity to fully understand the dynamics between CB and its effects. Finally, the research only addressed certain variables, excluding other possible influencing factors. Due to all of these limitations, cultural and social differences should not be overlooked.

### 4.2 Clinical and Policy Implications

First of all, it may be claimed that the findings from our study have many significant clinical and policy implications. The results of this research emphasize the importance of cooperation between different stakeholders to prevent CB and reduce its effects. Healthcare providers, policymakers, and families should always be mindful of the serious dangers that CB poses to young people (Suzuki et al. 2012). Continued research in this area and greater awareness may help to better protect adolescents against the negative effects of CB.

Schools, parents, teachers, and other adults play a crucial role in ensuring that adolescents are protected and supported against CB. Below are suggestions to prevent CB and reduce its effects. Schools are significant in preventing CB and reducing its effects. Schools can ensure that students have a safe learning environment by raising awareness against CB and developing educational programs. Teaching digital citizenship skills can help students demonstrate good behavior in the digital world and protect against harmful content. Additionally, schools can develop appropriate policies and procedures for early detection and intervention of CB.

Parents play an active role in protecting their children against CB. Parents can work to monitor their children's digital lives and understand what they will encounter online. By establishing open communication, children should be informed and supported about the effects of CB and how to cope with it. Parents must take on the responsibility of providing their children with satisfactory information about the risks of the digital world and the importance of using it safely. They should also teach their children skills to report CB and seek help and provide them with access to adults they trust. Teachers can undertake important duties to prevent and combat CB in the classroom environment. Teachers can work to teach students values such as empathy, respect, online ethics, and responsibility, and to explain the harms of CB. Through training programs, students should be provided with the skills to recognize, report, and intervene in CB. Finally, teachers can set classroom rules and procedures to ensure students stay safe online.

Moreover, when examining the effects of PR on adolescents, analyzing confounding variables such as socioeconomic status is important for enhancing the robustness of the study. Future research could explore the potential moderating or mediating role of socioeconomic status in the relationship between CB victimization and mental health outcomes. For example, the hypothesis that adolescents from lower socioeconomic backgrounds may have more limited resources to cope with the negative effects of CB, potentially weakening the protective effect of PR, could be tested. Such an analysis would help us better understand the effectiveness of PR interventions across different socioeconomic groups and assist in developing more targeted strategies.

## 5 Conclusion

As a result, it was found that CB was positively associated with DEP, ANX, and STR. Although CB did not have a direct effect on DEP and ANX, it had a direct effect on STR, which was partially mediated by PR. In summary, these findings suggest that interventions aimed at increasing PR may be effective in mitigating the negative impact of CB.

## Author Contributions

**Zafer Korkmaz;** Conceptualisation, Investigation, Writing ‐ original draft, Methodology, Validation, Visualisation, Project administration, Writing ‐ review & editing. **İlhan Çiçek**: Conceptualisation, Data curation, Investigation, Writing ‐ review & editing, Writing ‐ original draft, Methodology, Validation. **Metin Buluş**: Writing ‐ review & editing, Writing ‐ original draft, Software, Formal analysis, Visualisation, **Mehmet Emin Şanlı**: Writing ‐ review & editing, Project administration. **Murat Yıldırım**: Writing ‐ original draft, Validation, Writing ‐ review & editing, Supervision, Resources.

## Ethics Statement

The research was approved by the Batman University Ethics Committee (Code: 2023/02‐34). All procedures performed in studies involving human participants were in accordance with the ethical standards of the 1964 Helsinki Declaration and its later amendments or comparable ethical standards.

## Consent

Consent was obtained from all participants included in the study.

## Conflicts of Interest

The authors declare no conflicts of interest.

## Peer Review

The peer review history for this article is available at https://publons.com/publon/10.1002/brb3.70916

## Data Availability

The data supporting this study's findings are available from the corresponding author upon reasonable request.
